# Why training and specialization is needed for peer review: a case study of peer review for randomized controlled trials

**DOI:** 10.1186/s12916-014-0128-z

**Published:** 2014-07-30

**Authors:** Jigisha Patel

**Affiliations:** Biomed Central Ltd, Floor 6, 236 Gray’s Inn Road, London, WC1X 8HB UK

**Keywords:** Peer review, Evidence based medicine, EBM, Randomized controlled trials, RCT, Clinical training, Medical education, Reporting guidelines, CONSORT

## Abstract

**Background:**

The purpose and effectiveness of peer review is currently a subject of hot debate, as is the need for greater openness and transparency in the conduct of clinical trials. Innovations in peer review have focused on the process of peer review rather than its quality.

**Discussion:**

The aims of peer review are poorly defined, with no evidence that it works and no established way to provide training. However, despite the lack of evidence for its effectiveness, evidence-based medicine, which directly informs patient care, depends on the system of peer review. The current system applies the same process to all fields of research and all study designs. While the volume of available health related information is vast, there is no consistent means for the lay person to judge its quality or trustworthiness. Some types of research, such as randomized controlled trials, may lend themselves to a more specialized form of peer review where training and ongoing appraisal and revalidation is provided to individuals who peer review randomized controlled trials. Any randomized controlled trial peer reviewed by such a trained peer reviewer could then have a searchable ‘quality assurance’ symbol attached to the published articles and any published peer reviewer reports, thereby providing some guidance to the lay person seeking to inform themselves about their own health or medical treatment.

**Summary:**

Specialization, training and ongoing appraisal and revalidation in peer review, coupled with a quality assurance symbol for the lay person, could address some of the current limitations of peer review for randomized controlled trials.

## Background

### A brief history of trial reporting and peer review

‘Better have them all removed now.’ That was the advice I received in the early 1990s when my pain free un-erupted wisdom teeth first came to the notice of a surgeon. He was emphatic that I would suffer complications in the future if I did not have all four teeth removed under a general anesthetic. This seemed drastic to me, but I was given the same advice by two health professionals and it was with trepidation that I questioned their advice. At the time, ‘Evidence-Based Medicine’ which proposed the use of scientific evidence to inform clinical decision making was still a novel idea [1] and the Cochrane Collaboration [2], aimed at facilitating up-to-date systematic reviews of randomized controlled trials, had recently been founded.

I decided to search for the evidence. My only source of information was a medical library where I could identify and photo-copy relevant looking articles or get copies via an ‘inter-library loan’. I did not find any useful information, but I decided against the procedure on the basis that the risk of a general anesthetic and a stay in hospital seemed to me to completely outweigh any benefit of having four perfectly healthy pain-free teeth removed.

A short time later, when I was a junior doctor, a subgroup analysis of the diabetic patients who took part in the original ‘4S study’ [3], reported that simvastatin treatment improved morbidity and mortality in patients with diabetes [4]. At the time, my peers and I took for granted that the editors of the journals where the studies were published must have chosen the best people qualified to peer review and the peer reviewers must have done a competent job. The reported findings were compelling enough to have a profound effect on the care received by patients with diabetes.

These experiences not only illustrate the barriers to information I faced as a patient, but the power of individual clinical trials to directly influence treatment decisions for individual patients and the blind faith I and my peers had in a system whereby publication in a peer reviewed journal gave the reported results the status of ‘the evidence’ and, therefore, the ‘Truth’.

While my faith in the publication process was naïve and misplaced, flaws in the way randomised controlled trials (RCTs) were conducted and reported were recognised and initiatives were underway address these concerns. These culminated in the CONSORT statement [5] which aims to specify in detail how randomised controlled trials should be reported to improve transparency and help peer reviewers and readers make informed judgements about clinical trials. Since then a number of reporting guidelines for other types of clinical studies have been developed [6].

While my faith in the publication process was naïve and misplaced, flaws in the way RCTs were conducted and reported were recognized and initiatives were underway to address these concerns. These culminated in the Consolidated Standards of Reporting Trials (CONSORT) statement [5] which aims to specify in detail how RCTs should be reported to improve transparency and help peer reviewers and readers make informed judgments about clinical trials. Since then a number of reporting guidelines for other types of clinical studies have been developed [6].

While reporting guidelines aimed to address how individual trials were reported, there were also concerns about how far only positive or favorable findings were published while those with less exciting, favorable or inclusive findings were not (publishing bias). In 2005, the International Committee of Medical Journal Editors (ICMJE) published a statement announcing that its member journals would adopt compulsory trial registration as journal policy [7]. The aim was to register the existence of all clinical trials so that they became part of the public record.

Recently, in light of ongoing concerns about publication bias and the suppression of unfavorable results, the All Trials campaign [8] was launched which calls for the registering of all clinical trials and availability of all data for treatments in current use.

Meanwhile, running parallel with this, the world of peer review, was undergoing a revolution. Most definitions of peer review include a description of a process of scrutiny by independent experts or peers in the same field [9,10]. For peer-review journals this process involves sending submitted manuscripts to two or more people deemed to be knowledgeable enough in the field of the manuscript to judge its suitability for publication in that journal.

Flaws with the common single blind peer review system (where the reviewers know who the authors are, but the authors do not know who the reviewers are) were recognized [11] and there were experiments with double blind peer review to attempt to address this as well as in open peer review where the identity of reviewers and authors is known to all. While closed peer review did not appear to improve the quality of peer review [12], open peer review did appear to be feasible without undermining the quality of peer reviewer reports [13] and was first adopted by the *British Medical Journal* (*BMJ*) in 1999 [14].

The novel idea of an ‘Open Access’ journal, where all published research is freely available without subscription, began to emerge and although it was met by ferocious opposition from publishers [15], BioMed Central [16], the first completely online open access publisher was founded in 2000, followed, in 2006, by the launch of PLoS One [17].

The number of peer reviewed journals has been increasing at a steady rate of 3.5% a year and almost all are now available online [18]. With online publishing flourishing and with technical advances that allow comments to be made and shared in real time on a global stage, the process of traditional peer review, which can be slow and laborious, has been criticized [19]. New models of peer review have emerged and include (Table 1): re-review opt out [20], post-publication peer review [21], decoupled peer review [22-24], portable peer review [25], and collaborative peer review [26,27].

**Table 1:** **Models of peer review**

**Peer review model**		**Examples**	**Available information on peer review selection criteria**
Single blind	Reviewers know who the authors are, but authors do not know who the reviewers are.	The majority of biomedical journals	Varies from journal to journal. The journal editors select peer reviewers according to their own criteria.
Double blind	Both the reviewers and authors remain anonymous		As above
Open peer review	Both reviewers and authors are known to each other	First introduced by the *BMJ* [14]	As above
		BMC series medical journals [16]	
Re-review opt out	Authors are able to ‘opt-out’ of re-review after revisions if reviewers deem the research to be sound.	*BMC Biology*: [20]	As above, but one referee will usually be selected from those nominated by the author.
Collaborative peer review	Peer review includes a stage where the peer reviewers with or without the editor or authors take part in real time interactive discussion about the manuscript and agree a single set of revisions.	*Elife* [26]	A member of a ‘Board of Reviewing Editors’ oversees peer review and usually peer reviews themselves.
		*Frontiers* [27]	Members of the Editorial Board peer review and use a formal evaluation system
Portable peer review	Manuscripts which are peer reviewed by one journal, but rejected on grounds of threshold or interest are transferred together with their peer review reports to other journals which have the scope and threshold to match the manuscript. This can occur within a publisher or between a consortium of publishers.	BioMed Central [25]	Criteria for selecting peer reviewers will be that of the original journal
Decoupled peer review	Manuscripts are submitted to a peer reviewing service which organizes peer review and provides advice on appropriate journals based on the peer review reports.	Axios Review [23]	Criteria can vary. For example,
		Rubriq [24]	Rubriq: Peer reviewers must have a terminal degree in the area of interest, be employed full time in an accredited research university at the level of professor, instructor, post doc fellow or faculty research associate, must be a published first author or corresponding author in a peer reviewed academic journal within the last four years, and have prior experience as a journal peer reviewer. There is a standardized scorecard.
		Peerage of science [22]	
	Journals can also select manuscripts based on the peer review reports.		
			Peerage of science: Peer reviewers select the manuscripts they wish to review. Peer reviewers need to be scientists to qualify to peer review. Peer review reports are reviewed by fellow reviewers. Only scientists who have published a peer reviewed scientific article in an established international journal as first or corresponding author will be validated as Peers.
Post publication peer review	Manuscripts undergo initial checks and are published. Peer reviewers are then invited. Authors can revise their manuscripts. Revisions are published. If the manuscript ‘passes’ peer review, the article is indexed in databases such as Pub Med, Scopus etc	F1000Research [21]	F1000Research: Authors are asked to identify five potential referees who might be from the peer review panel. Author suggested referees should not have collaborated with the authors in the past five years, be from their own institution, or be too senior to be likely to undertake such refereeing (they should ideally have authored at least one article in the field as the lead author).

The impetus behind these recent initiatives has been to reduce delays for authors and reduce burden for reviewers. Their focus is on the process of peer review in terms of how and when it is done, rather than the substance and quality of peer review itself or expertise of the peer reviewer.

## Discussion

### The problem with peer review in medicine

Recent innovations in peer review seem to be driven by biologists with medical research ‘tagging along’. However, systems which might help biological research to thrive, might not necessarily be appropriate for research that directly influences patient care. There is no agreement on who a ‘peer’ or what ‘peer review’ actually is [11]. It is not clear what peer review aims to achieve [28] and no evidence that peer review works [29]. Journal instructions for peer reviewers [30] and the criteria for eligibility to peer review are variable (Table 1). There has been little evaluation of any of the more recent innovations in peer review for any outcomes. Furthermore, the whole system is based on honesty and trust and, as a consequence, is not designed to detect fraud.

Despite this, peer review is still seen by researchers as important and necessary for scientific communication [31] and publication in a peer reviewed medical journal is still the only valid or legitimate route to disseminating clinical research. In 2006, Richard Smith of the *BMJ* commented that it was, ‘odd that science should be rooted in belief’ [11]. In the world of evidence based medicine, it is astonishing that the evidence on which medical treatment is based is itself based on such precarious foundations with so many untested assumptions. Today, a junior doctor still relies on faith in the peer review system when judging a clinical trial and a patient searching, ‘Should I have my wisdom teeth removed if they don’t hurt?’ would get more than a million results on Google (search date 12 May 2014) with no guidance on the relevance or trustworthiness of any of them, leaving them as much in the dark as I was when I first asked that question. The difference between now and then is that then, information was just not available or accessible, and now, there is so much information available of varying quality that it is impossible to make sense of it all without some specialist knowledge. For example, if the lay person knows what to search for (prophylactic extraction of third molar) and which sources they can trust (the Cochrane library), the relevant information can be found easily. According to a Cochrane review I found [32], there is no evidence either way of the benefit of having wisdom teeth removed if they are asymptomatic. I feel reassured I made the right decision all those years ago. However, not all clinical questions can be answered so easily or can afford the luxury of waiting for a Cochrane systematic review to be done. When there is no ready-made Cochrane review, a system that provides some sort of quality check for individual studies might serve as an important consideration for patients (and doctors) who need to weigh up, using the available evidence, the risks and benefits of a course of action and make definitive, time dependent, decisions that could be life changing.

A UK Parliamentary enquiry on peer review in 2011 [33] concluded that different types of peer review are suitable for different disciplines and encouraged increased recognition that peer-review quality is independent of journal business model. With this in mind, is there a need to redesign peer review specifically for clinical research and ensure that this is driven by the clinical community?

### Training and specialization in peer review

With peer review as a vague and undefined process it is not surprising that in a survey of peer review conducted by Sense about Science, 56% of reviewers in a survey said there was a lack of guidance on how to review and 68% thought formal training would help [31]. Training and mentoring schemes for peer review have shown little impact [34-37] and even a decline in peer reviewer performance with time [38]. It may be that by the time a researcher has reached the stage in their career when they start to peer review, it is too late to teach peer review.

Although reporting guidelines have been available for two decades, many researchers and reviewers still do not understand what they are or the need for them. This is further compounded by inconsistent guidance from journals for authors on how to use reporting guidelines [30] and a lack of awareness of how they can improve the reporting of RCTs [39] and, thereby, aid peer review. There are misunderstandings about trial registration and even what constitutes an RCT. There is evidence that reviewers fail to detect deliberately introduced errors [34,37] and do not detect deficiencies in reporting methods, sometimes even suggesting inappropriate revisions [40]. Manuscripts reporting poorly conducted clinical research get published in peer reviewed journals and their findings inform systematic reviews, which in turn could also be poorly conducted and reported. These systematic reviews have the potential to inform clinical judgments.

The need for a concerted effort across disciplines to investigate the effects of peer review has been recognized [28], but before the effects can be investigated, the aims of peer review need to be defined. This is a daunting challenge if one aim, or a small number of aims, is intended to fulfill all peer review needs for all fields, specialties and study designs. A more manageable way may be to introduce specialization into peer review, so that specific fields can define the purpose and aims of peer review to suit their own needs and design training to meets those aims.

Since the methodology for conducting and reporting of RCTs has been defined by the CONSORT statement [41] which improves the reporting of RCTs [39] and, thereby, aids the peer review process, peer review of RCTs lends itself to such specialization. CONSORT could form the framework for the content of a training program and help to define the knowledge and skills that are needed by a given individual to appraise an RCT critically. Peer reviewers could be taught to spot fundamental flaws and be periodically evaluated to make sure they do, in the same way that any other knowledge or skill that affects patient care is.

**Figure 1:**
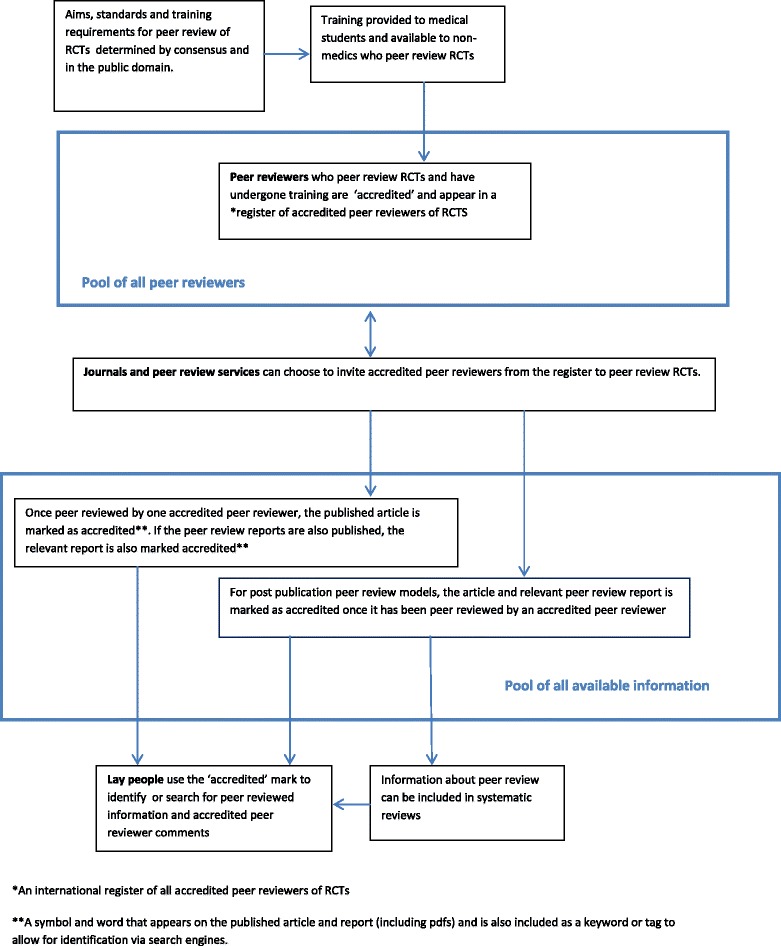
**Interaction of trained RCT peer reviewers with existing peer review models.** RCT, randomized controlled trial.

Peer review of RCTs should be recognized as a professional skill in this way. Every RCT, and its peer review reports if made public, whether published online, on paper, open access or subscription only, with open or closed peer review, or peer reviewed before or after publication could then have a searchable ‘quality assurance’ symbol (like the ‘kite-mark’ used by the British Standards Institute [42]) or a word, so that readers know whether a study was reviewed by at least one appropriately trained and accredited expert. Such a system could accommodate all peer review models (Figure 1).

To achieve this, major organizations including medical schools, medical regulatory and accreditation organizations (such as the General Medical Council and Royal Colleges in the UK), funding bodies, publishers and journal editors and lay people need to come to a consensus on the definition, purpose, standards and training requirements of peer review of RCTs. Training should begin in medical schools and be ongoing.

By recognizing peer review as a professional skill with measurable standards which are separate from the journal, publisher or peer review model, peer review is separated from commercial considerations, peer reviewers get recognition for their work, and researchers, clinicians and patients get some indication of quality on which to base their judgments. Publishers and journals are then free to innovate while still maintaining consistency of peer review for RCTS, editors have clear criteria on which to base their choice of peer reviewer for a given manuscript and a baseline is set that allows for future research into the effectiveness of peer review *per se* and comparative studies on the effectiveness and quality of emerging innovations.

## Summary

While innovations in trial reporting and the peer review process have increased transparency, there has been little progress in defining the aims and effects or improving the quality of peer review itself. There is a vast volume of health information available to the lay person with little or no guidance on its quality or trustworthiness.

Treatment decisions are based on evidence which is itself determined by a system for which there is no evidence of effectiveness. Innovations in peer review that specifically address the quality of peer review and the expertise of the peer reviewer and provide guidance for lay people seeking to inform themselves about their own health related decisions are urgently needed. Formal professional training for peer review of RCTs coupled with a means of identifying RCTs peer reviewed by such trained experts could address these needs.

The focus of this article has been on peer review of evidence-based medicine and RCTs in particular because the consequences of an ill-defined system of peer review are easily understandable by the scientist and the lay person alike. However, the purpose of peer review and a method of training and evaluating peer reviewers could be defined in a similar way for any other type of study design or any other field.

## Abbreviations

CONSORT: The Consolidated Standards of Reporting Trials
RCT: randomised controlled trial
